# Emerging Effects of Sepantronium Bromide (YM155) on MOLT-4 Cell Line Apoptosis Induction and Expression of Critical Genes Involved in Apoptotic Pathways

**DOI:** 10.15171/apb.2020.010

**Published:** 2019-12-11

**Authors:** Kobra Shojaei Moghadam, Majid Farshdousti Hagh, Mohammad Reza Alivand, Masoumeh Fardi, Ali Akbar Movassaghpour, Ali Mohammadi, Maryam Moghadasi, Saeed Solali

**Affiliations:** ^1^Student Research Committee, Tabriz University of Medical Sciences, Tabriz, Iran.; ^2^Immunology Research Center, Tabriz University of Medical Sciences, Tabriz, Iran.; ^3^Drug Applied Research Center, Tabriz University of Medical Sciences, Tabriz, Iran; ^4^Department of Medical Genetic, Tabriz University of Medical Sciences, Tabriz, Iran.; ^5^Hematology and Oncology Research Center, Tabriz University of medical Sciences, Tabriz, Iran.; ^6^Departments of Cancer and Inflammation Research, Institute of Molecular Medicine, University of Southern Denmark, Odense, Denmark.; ^7^Molecular Medicine Research Center, Tabriz University of Medical Sciences, Tabriz, Iran.; ^8^Division of Hematology and Transfusion Medicine, Department of Immunology, Faculty of Medicine, Tabriz University of Medical Sciences, Tabriz.

**Keywords:** Apoptosis, Cancer, Drug, Survivin, MOLT-4, YM155

## Abstract

***Purpose:*** Sepantronium bromide (YM155) is a Survivin inhibitor which recently advanced as an anticancer agent in phase II clinical trials. Survivin belongs to IAP (inhibitor of apoptosis) gene family and is a pivotal target for treatment due to its overexpression and oncogenic function in many malignancies, including acute lymphoblastic leukemia (ALL). Although survivin is a specific target for YM155, recent reports have shown that it has many other crucial targets that regulate its anti-apoptotic effects. The aim of this study was to investigate whether YM155 could have an effect on cell death-inducing genes as well as inducing apoptosis in T-ALL MOLT4- cell line.

***Methods:*** We treated MOLT-4 cells with increasing concentrations of YM155 and then cell viability was determined using MTT (methyl thiazolyl tetrazolium) assay. Also, the rate of induction of apoptosis in MOLT-4 cells and the target genes expression levels were evaluated by Annexin V/PI and real-time PCR, respectively.

***Results:*** YM155 inhibited cell growth in MOLT-4 cells. This outcome is achieved by inducing apoptosis and a significant increase in the expression level of P53, MiR-9, caspase 3 and decreasing the mRNA expression levels of survivin, Sirtuin1(SIRT1), member of anti-apoptotic proteins family (Bcl-2), and epithelial-to-mesenchymal transition (EMT) initiating factors Snail1and Zeb2.

***Conclusion:*** The results showed that use of YM155 can be a potential drug therapy in T-ALL patients with promising effects on apoptosis induction.

## Introduction

Acute lymphoblastic leukemia )ALL) is one of the most common variants of leukemia, which affects all ages, with high costs and treatment failure.^1^ Although the diagnosis and treatment of these malignancies nowadays have greatly improved, the overall survival rate of patients is still low.^2^ Therefore, in order to reduce the therapeutic burden, target therapy in ALL patients seems essential. For this reason, in recent clinical and preclinical studies, many targets have been tested for effective treatment.^2^

YM155 with the original name of 1-(2-methoxyethyl)-2-methyl-4,9-dioxo-3-(pyrazin-2-ylmethyl)-4,9-dihydro-1H-naphtho [2,3-d] is a pioneer chemical compound with significant therapeutic effects, which has been shown to induce apoptosis, inhibit invasion and suppress drug resistance in numerous cancer cell lines.^3-8^ YM155 has been shown to be tolerable in phase II clinical trials in patients with melanoma^8^ and advanced refractory NSCLS.^9^

It has been reported that YM155 applies its anti-tumor property through direct suppression of *survivin* expression by binding to its promoter.^3^

*Survivin*, which is the smallest member of the IAP family, is overexpressed during the development of human cancer and is associated with poor survival.^10^ In cancer cells, upregulation of *survivin* leads to the inhibition of apoptosis via interaction with caspase3 and 7 to inhibit their enzymatic activity.^11^ In addition, it has been demonstrated that overexpression of *survivin* in primary ALL plays a critical role in drug resistance and its down-regulation in combination with chemotherapy leads to the eradication of ALL leukemia cells.^10^

Studies have shown that YM155 causes cell death by different mechanisms other than suppression of *survivin*; for instance, it is able to arrest the cell cycle in the S phase and as a result it can evoke DNA damagecascades.^12^ Also, it was documented that YM155 reduced the resistance to treatment through reversing a process called epithelial-to-mesenchymal transition (EMT) by inhibiting the STAT3 pathway.^13^

EMT is a crucial mechanism during cancer progression by changing the epithelial state of the cells to mobile and mesenchymal-like state. This process is accompanied by acquiring many oncogenic properties for the tumor cells, including apoptosis resistance, proliferation, differentiation arrest and invasion of cancer cells.^14-16^ A study found that the use of YM155 reduces the EMT-inducing transcription factor called Zeb1 (zinc-finger E-box-binding) via the STAT3 pathway.^13^ However, the effect of this agent on other EMT-inducing factors, including Zeb2 and Snail1, has not yet been studied.

It has also been shown that the use of YM155 is associated with a significant reduction in the expression of member of anti-apoptotic proteins family (Bcl-2) in the HL-60 cell line, but its effects on the U937 cell line was not significant. In addition, this study examines the role of YM155 in the induction of caspases. The results indicate that the use of this inhibitor, in contrast to the U937 cell line, is able to increase caspases expression in the HL-60 cell line.^17,18^ We also investigated the expression levels of Bcl2 and caspase3 in MOLT-4 cell line treated with YM155.

Furthermore, a whole transcription study on MDA-MB-231 cells after YM155 treatment documented that this drug also changed the expression pattern of micro-RNAs such as miR-125b-1.^19^ In the present study, we studied the role of YM155 on the induction of apoptosis and gene expression associated with the cell death and apoptosis; we also defined the expression of miR-9 as an important regulator of gene expression in T-ALL MOLT4 cell line.

## Materials and Methods

### Cell culture

We used the MOLT-4 cell line, purchased from the cell bank of the Pasteur Institute of Tehran (Iran). The cells were then grown under standard conditions suitable for their growth, including culture media, RPMI-1640, plus 10% fetal bovine serum (FBS) and 100 mg/mL of penicillin and streptomycin antibiotics (all purchased from Gibco Inc.). Then the cells were incubated in 5% carbon dioxide (CO_2_) at 37°C.

### Evaluation of cytotoxic effect of YM155 on MOLT4 cells

YM155 was purchased from Sigma Aldrich (Cat Number 781661-94-7), dissolved in dimethyl sulfoxide (DMSO) with 225mM stock solution and stored at −20°C.

This test is based on the activity of the mitochondrial succinate dehydrogenase enzyme in living cells by measuring their optical absorption. The cells were cultured with an initial number of 1×10^5^cells/well in each well of a 96-well plate. After 24 hours, the cultured cells were treated with different concentrations of YM155 (0–6 mM). Twenty-four and 48 hours after treatment, 25 μL of MTT solution was added to the cells and the cells were incubated for 4 hours. We then centrifuged the plate to re-precipitate the cells. Then we dispersed the supernatant. To each well, 200 μL of MTT solubilizing solution (containing DMSO and Sorenson buffer) were added. After 30 minutes of incubation of the cells under the above conditions, optical densities of the wells were read at 570 nm with an ELISA reader.

### Apoptosis study using flowcytometry

In order to determine the percentage of apoptotic cells treated with YM155 and compare with the control cells’ population, staining of cells with two stains, Annexin V and propidiumiodide (PI), was performed using the Apoptotest™ FITC kit (EXbio). First MOLT4 cells were treated for 24 and 48 hours with the IC50 concentration of YM155; subsequently, the cells were multiple-washed with PBS buffer and the pellets were re-suspended in 100 μL of the binding buffer.

The cells were incubated with 20 μL of PI and 10 μL of Annexin V labeled with FITC at room temperature in a dark environment for 10 minutes. Finally, the study of apoptosis in MOLT-4 cells treated with YM155 was evaluated by FACSCalibur (BD Biosciences, Franklin Lakes, NJ) and FlowJo software version 10.4.1 (Treestar, FlowJo).

### RNA extraction

Extraction of total mRNA from MOLT-4 cells was carried out 24 hours after treatment with YM155 by using RiboEx reagent (Gene All, South Korea), according to the manufacturer’s instructions.

### Quantitative real-time PCR (qPCR) for miRNA and mRNA expression

All the primers used in this study were designed by Oligo7 software and then blasted with the NCBI site by Primer-Blast (Table 1). Briefly, 1 µg in 20 µL of total volume RiboEX extracted RNA was reversely transcribed into cDNA system-loop primer or oligo (dT) using the Script™ First-µ Strand Synthesis System (Invitrogen) and incubated for 60 minutes at 42°C and 5 minutes at 70°C, followed by enzyme heat-inactivation.

**Table 1:** List of primers used in this research

**Name**		**Sequence**
Survivin	Forward	ATGGGTGCCCCGACGTTGCC
	Reverse	GCTCCGGCCAGAGGCCTCAA
miR9	Forward	ATTTCTGCCAGGACCGCTTCTAC
	Reverse	ATCCGGCAAACTGGCTCCTTC
MDR1	Forward	TCACTATTGTTTCTAGCCCTT
	Reverse	CTTTGCCAAATGTGAAACCC
SIRT1	Forward	GCCTCACATGCAAGCTCTAGTGAC
	Reverse	TTCGAGGATCTGTGCCAATCATAA
Snail1	Forward	ACTATGCCGCGCTCTTTCCT
	Reverse	GCTGCTGGAAGGTAAACTCTGG
P53	Forward	GCCATCTACAAGCATGCACAGCA
	Reverse	GTCATTCCAAATACTCCACACGCA
Caspase3	Forward	AAGCGAATCAATGGACTCTGG
	Reverse	CTGTACCAGACCGAGATGTC
Bcl-2	Forward	GCTGCACAAATACTCCGCAAG
	Reverse	TGCCAAATCTTCGGAGACGAC
Apex1	Forward	CTTTAGGCACCTCTACCCCAA
	Reverse	CGAGCATTCATCATATAAGTCCA
Zeb2	Forward	CTTGCCCCTCCTGTTACCC
	Reverse	CAGCCCTAATGTGCAATCGT
U6	Forward	GCTTCGGCAGCACATATACTAAAAT
	Reverse	CGCTTCACGAATTTGCGTGTCAT
β-actin	Forward	GGAGTCCTGTGGCATCCACG
	Reverse	CTAGAAGCATTTGCGGTGGA

Synthesis of cDNA was performed using a Thermo Scientific Revert Aid First Strand cDNA Synthesis kit and 500 ng of extracted RNA.

Evaluation of the expression of genes was performed by qRT-PCR using SYBR Green master mix (Exiqon, Vedbæk, Denmark) in the Roche Light Cycler 96 system (Roche, Germany). β-actin was used as an internal control for the evaluation of mRNAs expression. In addition, we used U6 as a reference gene for the evaluation of miRNA expression.

### Statistical analysis

Data were analyzed with GraphPad Prism version 8.0.2 for Windows. Data were presented as M ± SEM (mean ± standard error of the mean). *P*< 0.05 (*) and *P*< 0.01 (**) were considered statistically significant. Also, flowcytometry data were analyzed using FlowJo^®^ software (Treestar, Inc).

## Results and Discussion

### YM155 has a cytotoxic effect on MOLT4 cells

**Figure 1:**
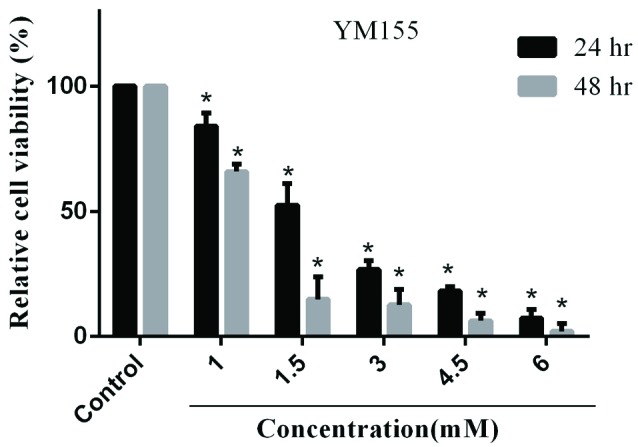
In vitro anti-proliferative activity of YM155 against MOLT4 cell line. YM155 reduced the viability of the MOLT-4 cells as determined by MTT assay with the presence of various doses of YM155 (0–6 mM) for 24 and 48 hours of incubation.

To assess the cytotoxic effect of YM155 on the viability of MOLT-4 cells, they were treated with different increasing concentrations of YM155 (0 to 6 mM for 24 and 48 hours) and evaluated using MTT test. The mean IC50 was 1.82 mM for 24 hours (Figure 1).

### YM155 induced cell death through apoptosis in MOLT4 cells

**Figure 2:**
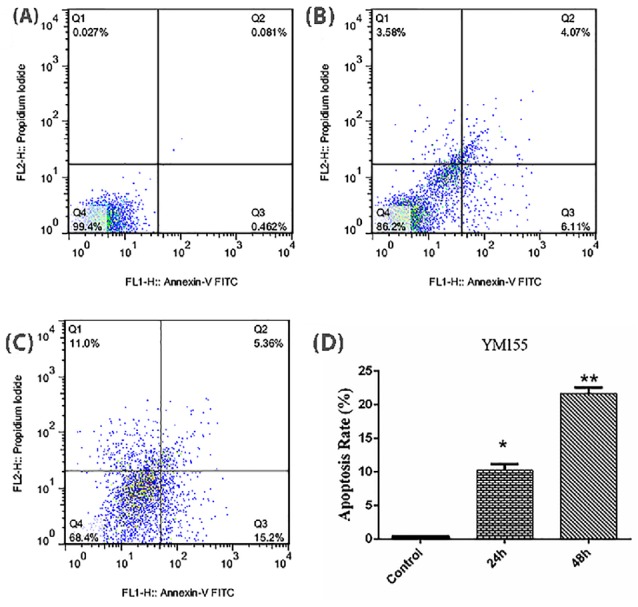
YM155 induced apoptosis in human leukemia MOLT4 cell line by using Annexin V staining and PI. (A) The cells were incubated with 0.1% dimethyl sulfoxide (DMSO) as a control group. (B) MOLT4 cells were incubated with 1.82-mmol YM155 for 24 hours. (C) MOLT-4 cells that were incubated with 1.82-mmol YM155 for 48 hours. (D) The percentage of apoptosis in three groups: A, B and C; *P≤0.05; **P≤0.01. PI: propidium iodide.

To study the effect of YM155 on apoptosis in MOLT4 cells in vitro, we prepared MOLT4 cells for apoptosis analysis via Annexin V-FITC, following treatment with YM155 (1.82 mM) for 24 and 48 hours. The results showed that YM155 was able to augment apoptosis in MOLT4 cells in a time-dependent manner compared to the untreated group (Figure 2).

### YM155 increased miR-9 and decreased survivin, Bcl-2 and genes related to EMT and DNA repair expression level

**Figure 3:**
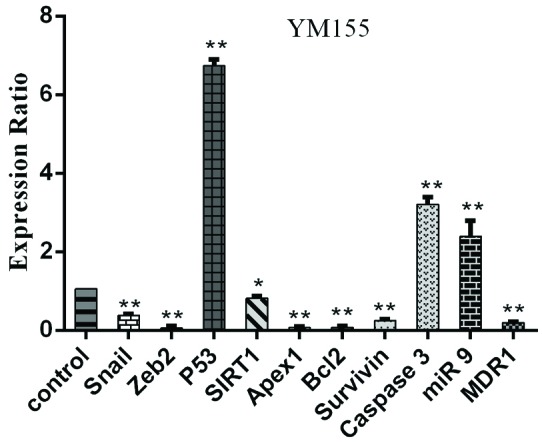
Changes in the expression of *survivin*, Snail1, Zeb2, P53, SIRT1, Apex1, Bcl-2, caspase3, miR-9 and MDR1in MOLT-4 cell line treated with the IC50 concentration of YM155 compared to a control group (untreated cell line) (*P<0.05, **P<0.001).

We used a qRT-PCR to evaluate *survivin* and several other candidate genes along with miR-9 expression in MOLT-4 cells treated with YM155 (1.82 mM) for 24 hours. It was observed that YM155 decreased the expression of genes associated with apoptotic inhibition such as *survivin*, Zeb2, Snail1, SIRT1, MDR1, Apex1 and Bcl-2 significantly. It also caused an increase in the expression of caspase3, P53, and miR-9. To determine differentially expressed miR-9, *survivin*, Bcl-2, caspase3 and other genes, the ANOVA results of normalized data generated (at *P*<0.05) between the group with YM155 treatment and the control group (no treatment) are shown in Figure 3.

In this study, we showed that YM155 inhibits MOLT-4 cell growth. Treatment of MOLT4 cells with different concentrations of YM155 led to the inhibition of proliferation and induction of apoptosis. In fact, we showed that in addition to s*urvivin*,YM155 down-regulated Bcl-2,which consequently resulted in elevated expression of caspase3 as an apoptosis inducer.

YM155 is a dioxonaphthoimidazolium analog that is the first drug reported to inhibit *survivin* expression.^20^ *Survivin* as an inhibitor of apoptosis is encoded by the BIRC5 gene with crucial functions in the regulation of apoptosis, cell division and cellular stress responses.^21^ *Survivin* is markedly expressed in most malignancies, including leukemia, and is associated with poor prognosis.^22^ Previous studies have demonstrated that *survivin* can promote drug resistance through both the stabilization of microtubule organization and co-working with other drug resistance molecules. Wang et al showed that patients with AML that exhibit overexpression of *survivin* and MDR1 have poor therapeutic response and survival.^23^ *Survivin* is also associated with DNA double-strand break (DSB) repair machinery and increases the repair process.^24,25^ These findings suggest that *survivin* is involved in various intracellular events in cancer cells via interactions with other molecules. However, it seems that the precise mechanism of induction of the above-mentioned processes needs to be elucidated. Studies have shown that YM155 significantly suppresses the growth of various cancer cell lines and xenograft models, including breast cancer, hormone-refractory prostate cancer, ovarian cancer, leukemia and hepatoma through reducing the expression of *survivin*.^20,26-28^ In line with previous findings, our results showed that YM155 effectively reduced *survivin* mRNA expression level in the T-ALL cell line.

YM155 has been demonstrated to not only inhibit *survivin*, but also regulate expression of a large number of genes, including X Chromosome-linked inhibitor of apoptosis (XIAP), Zeb1 and anti-apoptotic protein (Mcl-1), various micro-RNAs and DNA damage repair genes,^13,18,19,29-32^ suggesting that anti-tumor function of YM155 is beyond the inhibition of *survivin.*

Micro-RNAs are a group of non-coding RNAs with a length of 21–25 nucleotides, which play important roles in the regulation of expression of target genes through targeting (3’-UTR) mRNAs via base pairing.^33^ Several studies have described regulatory functions for miR-9 in the chemo-responsiveness of leukemic cells. For example, Zang et al, by analyzing qRT-PCR, measured miR-9 gene levels in vitro, including MOLT-4 cells, and showed that expression of miR-9 was significantly lower than non-leukemic primary peripheral blood control cells. Furthermore, by using lentiviral transduction, they generated miR-9 over-expressed cell lines and examined their proliferation. During this study, the phases G0 and G1 were prolonged, but phase S was shortened. This finding showed that increased expression of miR-9 by anti-malignant activity inhibited proliferation and cell cycle in acute lymphoblastic leukemia cells.^34^ Yanli et al confirmed that multidrug resistance 1 (MDR1) is a negative target for miR-9 in chronic myeloid leukemia and observed that inhibition of miR-9 decreased the chemo sensitivity‍of K562 cells by overexpression of MDR1.^33^ In another study, Zhou et al reported that miR-9 directly targeted and regulated Sirtuin1 (SIRT1) which is an important regulator of cellular stress response and genomic integrity.^35^ SIRT1 is overexpressed in most cancers such as T-ALL.^36,37^ It has been shown that SIRT1 inhibits apoptosis through deacetylation and decreasing the transcriptional activities of the protein substrates, including of tumor suppressor p53, because acetylation of p53 results in increased expression of downstream effector such as P21, Bax and PUMA which are involved in inducing apoptosis. Therefore, it can be concluded that SIRT1 indirectly inhibits apoptosis by inhibiting p53.^37^ Jang et al found that SIRT1 inhibition increases DNA damage by reducing the level of DNA repair enzymes such as Apex1 and results in the activation of p53, leading to cell death through the activation of a number of genes involved in apoptosis.^38^

**Figure 4:**
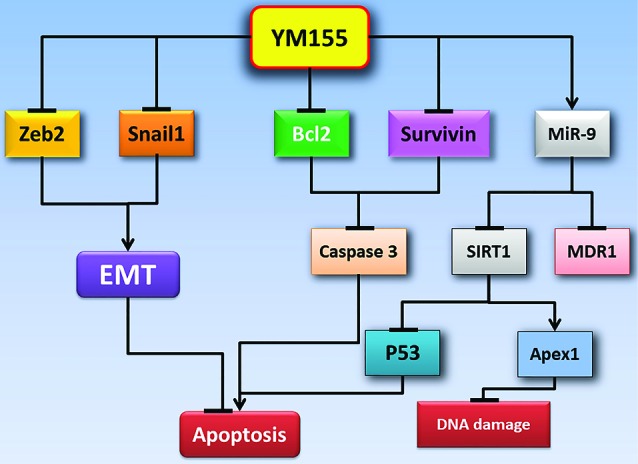
Schematic pathways of YM155 effects on the expression of selected genes in MOLT-4 cell line with q-RT PCR analysis.In this study, we found that *survivin* and Bcl2 genes were inhibited by YM155, and as a result, the expression of Caspase3 increased. It was also found that MiR-9 expression was induced with this agent. The result is inhibition of the expression of SIRT1 and MDR1. In addition, YM155 inhibited the expression of EMT-inducing factors (Zeb2, Snail1).

This study showed that after 24 hours of treatment with YM155, the miR-9 expression levels increased significantly compared to control cells; in addition, the expression of two target genes, including MDR1 and SIRT1, decreased significantly. It is suggested that one of the effects of YM155 on inhibiting the growth and DNA damage response of leukemic cells can be via the miR-9/SIRT1/p53 pathway (Figure 4). Furthermore, a decrease in the expression of MDR1 can indicate the effectiveness of YM155 in increasing the sensitivity of cancer cells to chemotherapy drugs and reducing drug resistance.

EMT can be defined by the loss of cell–cell adhesion by down-regulation of the adhesion molecule E-cadherin and up-regulation of mesenchymal markers, such as N-cadherin. Upon this process, cancer cells are able to increase their mobility and invasiveness during cancer progression.^39,40^

Different transcription factors, including the Snail1 and Zeb2, serve as molecular regulators of the EMT process. Several studies have demonstrated that low levels of Zeb2 and Snail1 contribute to apoptosis induction. While increased expression of these genes contributes to tumor progression by protecting cancer cells from apoptosis via various processes such as repressing the expression of caspase3; therefore, one of the features of EMT in cancer cells is the inhibition of induction of apoptosis by increasing the expression of Zeb2 and Snail1.^15,16,41,42^ Studies have shown that YM155 inhibits EMT in cancer cells.^13,28^ Zhang et al found that YM155 treatment leads to increased levels of E-cadherin but decreased N-cadherin by targeting STAT3 in glioblastoma cells.^13^ STAT3 is a transcription factor that acts as a regulator to control the expression program of tumor-associated genes, including Snail and Zeb2.^14,43^ This study showed that YM155, in addition to *survivin* inhibition, reduces the expression of Zeb2 and Snail1, possibly, indicating that one of the major ways that YM155 induces apoptosis is through suppressing EMT-TFs like Snail1 and Zeb2.

## Conclusion

The results of the present study showed that YM155 can inhibit MOLT-4 leukemia cell line growth and induce apoptosis by down-regulation of critical genes involved in apoptosis and cell death, including *survivin*, Bcl-2, Apex1, Zeb2, Snail1, SIRT1 and up-regulation of miR-9, caspase3 and p53 in MOLT-4 cell line. It suggests that YM155 could potentially be an effective therapeutic strategy for further investigations into leukemia.

## Ethical Issues

Not applicable.

## Conflict of Interest

Authors declare no conflict of interest in this study.
